# Versatile biotechnological applications of amylosucrase, a novel glucosyltransferase

**DOI:** 10.1007/s10068-019-00686-6

**Published:** 2020-07-10

**Authors:** Dong-Ho Seo, Sang-Ho Yoo, Seung-Jun Choi, Young-Rok Kim, Cheon-Seok Park

**Affiliations:** 1 Department of Food Science and Technology, College of Agriculture and Life Sciences, Jeonbuk National University, Jeonju, 54896 Republic of Korea; 2 Department of Food Science and Biotechnology, and Carbohydrate Bioproduct Research Center, Sejong University, Seoul, 05006 Republic of Korea; 3 Department of Food Science and Technology, Seoul National University of Science and Technology, Seoul, 01811 Republic of Korea; 4 Graduate School of Biotechnology and Institute of Life Science and Resources, Kyung Hee University, Yongin, 17104 Republic of Korea

**Keywords:** Amylosucrase, Transglycosylation, Enzymatically modified starch, Turanose, Amylose

## Abstract

Amylosucrase (AS; EC 2.4.1.4) is an enzyme that has great potential in the biotechnology and food industries, due to its multifunctional enzyme activities. It can synthesize α-1,4-glucans, like amylose, from sucrose as a sole substrate, but importantly, it can also utilize various other molecules as acceptors. In addition, AS produces sucrose isomers such as turanose and trehalulose. It also efficiently synthesizes modified starch with increased ratios of slow digestive starch and resistant starch, and glucosylated functional compounds with increased water solubility and stability. Furthermore, AS produces turnaose more efficiently than other carbohydrate-active enzymes. Amylose synthesized by AS forms microparticles and these can be utilized as biocompatible materials with various bio-applications, including drug delivery, chromatography, and bioanalytical sciences. This review not only compares the gene and enzyme characteristics of microbial AS, studied to date, but also focuses on the applications of AS in the biotechnology and food industries.

## Introduction

Amylosucrase (AS, E.C. 2.4.1.4) is a versatile sucrose-hydrolyzing enzyme that belongs to the glycoside hydrolase (GH) family 13; it was discovered by Hehre and Hamilton from *Neisseria perflava,* in 1946 (Hehre and Hamilton, 1948). The uniqueness of this enzyme lies its ability to synthesize an amylose-like polysaccharide directly from sucrose, without the addition of an α-d-glucosyl-nucleotide-diphosphate, like ADP- or UDP-glucose (Hehre et al., 1949; Okada and Hehre, 1974). It was found that AS was activated by exogenous amylopolysaccharides such as glycogen and that the linear α-glucan polysaccharides synthesized by AS did not have α-1,6-linked glucosyl residues. The degree of polymerization (DP) of the α-1,4-linked glucans was in the range of 2–55, depending on the origin of the microorganism (Potocki-Veronese et al., 2005). This polymerization reaction was observed to be inhibited by high concentrations of sucrose (> 100 mM), and an alternative isomerization reaction was stimulated, leading to the production of sucrose isomers, such as turanose (Wang et al., 2012).

Recently, the transglycosylation activity of AS was extensively studied using exogenous bio-functional compounds other than amylopolysaccharides, such as flavonoids, as glycosylation acceptor molecules. It was found that a diverse group of biological compounds could serve as acceptors in the transglycosylation reaction of AS. It transferred one or several glucose molecules to its acceptor compounds, which resulted in their increased solubility and bioavailability (Lee et al., 2017b; Yamada et al., 2006). The advantages of using AS for intermolecular transglycosylation are therefore its broad spectrum acceptor specificity and the requirement of inexpensive substrate. This means that AS can be used as a special tool for enzymatic transglycosylation in various biotechnological fields. The application of AS is not limited to the production of amylose-like polymers, but also extends to the synthesis of functional carbohydrates, including low digestive modified starches, trehalose, turanose, and the biosynthesis of glucosyl bioactive compounds. In addition, the application of AS was expanded to the biosynthesis of amylose microparticles by utilizing the amylose-like polymer production capacity. In this review, the characteristics of various microbial ASs are discussed and the most recent applications of AS in food are summarized, along with their possible uses in the future.

## Discovery and enzymatic properties of various microbial AS

In 1997, the gene coding for AS in *Neisseria polysaccharea* ATCC 43768 (*npas*) was first cloned into an inducible expression system in *Escherichia coli* (Büttcher et al., 1997). The recombinant enzyme was reported to synthesize an amylose-like polymer from sucrose. However, incorrect information for the *npas* gene was published. After 2 years, other research groups reported the exact same information for the *npas* gene (gene locus_tag: AJ011781.1 protein ID: CAA09772.1) (Potocki de Montalk et al., 1999). NpAS was expressed as a fused protein with glutathione S-transferase in *E. coli* and was easily purified by affinity chromatography. The purified recombinant NpAS could linearly elongated some branched chains of glycogen (Rolland-Sabaté et al., 2004). Advances in whole genome DNA sequencing technology have led to the discovery of the genes encoding putative AS genes from various microorganisms. Recently, the AS gene from *Neisseria subflava* ATCC 49275 (gene locus_tag: NEISUBOT_05048, protein ID: EFC51554.1, *nsas*) was cloned, based on the sequence of the *npas* gene and its expression and enzyme characteristics were confirmed (Park et al., 2018a). Most microbial AS genes in the early 2000s were not annotated with the term, “amylosucrase”. The enzymatic properties of the α-amylase encoding genes (gene locus_tag: NC_001263.1, protein ID: NP_294657.1, *dras*) that were annotated in *Deinococcus radiodurans* ATCC 13939 genome did not show α-amylase, but instead showed AS. The DrAS shares 42% amino acid identity with NpAS and displays typical AS activity such as sucrose hydrolysis, transglycosylation (or polymerization), and isomerization, using sucrose as the sole substrate (Pizzut-Serin et al., 2005). The whole genome sequence of *Deinococcus geothermalis,* which belongs to the same genus as *Deinococcus radiodurans,* was completely reported by DOE (U.S. Department of Energy) in 2007 (Makarova et al., 2007). Although the ORF Dgeo0572 (gene locus_tag: CP000359.1, protein ID: ABF44874.1, *dgas*) was annotated with an α-amylase encoding gene, it was confirmed as an AS by Seo et al. (2008). In the whole genes of *D. radiopugnans* ATCC 19172, two sucrose phosphorylase genes were annotated through RAST server analysis (unpublished). The amino acid sequence of one of these sucrose phosphorylase genes (gene locus_tag: MK766972, protein ID: QCT05769, *drpas*), was 76 and 74% similar to the amino acid sequences of DgAS and DrAS, respectively, and AS activity was detected when the protein was expressed in *E. coli* (Kim et al., 2014c; 2014d). Two AS genes from *Alteromonas* species (*Alteromonas addita* KCTC 12195, gene locus_tag: AB469415.1, protein ID: BAG82877.1, *aaas,* and *Alteromonas macleodii* KCTC 2957, gene locus_tag: AB469558.1, protein ID: BAG82876.1, *amas*) were cloned, sequenced and expressed in *E. coli*. AaAS and AmAS were 48 and 49% similar to NpAS and showed 77% amino acid homology with respect to each other (Ha et al., 2009). Interestingly, the recombinant AmAS displayed typical AS activity, whereas the recombinant AaAS did not utilize the sucrose as a substrate. *Synechococcus* sp.—that is very widespread in marine environment—like the *Alteromonas* species, has a sucrose utilizing cluster containing sucrose phosphate synthase, sucrose-phosphate phosphatase, fuctokinase, and AS. The AS from *Synechococcus* sp. PCC 7002 (gene locus_tag: FXWN01000001.1, protein ID: SMQ77851.1, *syas*) displayed typical AS activity such as hydrolysis and transglycosylation actives, and its transcription was increased after a salt-treatment (Perez-Cenci and Salerno, 2014). An *acas* (gene locus_tag: CP001341.1, protein ID: ACL41561.1) designated as an α-amylase was cloned from *Arthrobacter chlorophenolicus* A6, a gram-positive *Actinobacterium* capable of surviving under psychrophilic conditions. The gene product was found to have AS activities, and not α-amylase activities (Seo et al., 2012a). The *Neisseria*, *Deinococcus*, *Alteromonas*, *Arthrobacter,* and *Synechococcus* species are known as the microorganisms that form biofilms to survive in harsh conditions. The biofilm is a complex polymer composed of extracellular DNA, protein, and polysaccharides. The AS is probably related to the synthesis of biofilm in these microorganisms. However, the AS gene is also found in microorganisms that do not form biofilms. *Methylobacillus flagellatus* KT and *Methylomicrobium alcaliphilum* 20Z, which can utilize methane, have sucrose utilizing clusters similar to *Synechococcus* sp.; *mfas* (gene locus_tag: CP000284.1, protein ID: ABE50875.1) and *maas* (gene locus_tag: FO082060.1, protein ID: CCE22312.1) from *Methylobacillus flagellatus* KT, and *Methylomicrobium alcaliphilum* 20Z, respectively, were successfully expressed in *E. coli* and showed typical AS activity (But et al., 2015; Jeong et al., 2014). Recently, *ccas* (gene locus_tag: AXCY01000026.1, protein ID: KGM11272.1) and *btas* (gene locus_tag: BTHE_RS02440, protein ID: WP_044279707.1) genes from *Cellulomonas carboniz* T26 and *Bifidobacterium thermophilum* ATCC 25525, respectively, were cloned in *E. coli* through sequence analysis, and each enzyme showed typical AS activity (Choi et al., 2019; Wang et al., 2017). Summary information and comparisons of the cloned AS genes are presented in Table 1. The enzyme characteristics of the microbial AS were studied with recombinant protein expression in *E. coli*. In most studies, the optimal temperature, pH, and enzyme units were measured using the simple 3,5-dinitrosalicylic acid (DNS) method to measure the ability of AS to utilize sucrose as a sole substrate. There is a difference in the enzyme measurement methodologies between research groups, and it is thus difficult to compare the exact AS activity levels. This review discusses the activity of AS when the activity were measured by the same experimental method. Most of the AS showed optimum enzyme activities in neutral conditions of pH 7.0–8.0 (Table 1). Interestingly, the BtAS present in *Bifidobacterium thermophilum* cultured under acidic conditions, showed optimal activity at weakly acidic conditions of pH 6.0 (Choi et al., 2019). The optimum temperature for microbial AS activity was 30–40 °C, except for DgAS and BtAS, for which the optimum temperature was 50 °C (Choi et al., 2019; Seo et al., 2008). In addition, these two enzymes showed higher thermal stability at 50 °C than the other microbial ASs. The thermal stability of enzymes is an important parameter for industrial application, and some researchers have conducted studies to improve the thermostability of NpAS through directed evolution (Emond et al., 2008). The half-life of the double-mutant NpAS, R20C/A451T at 50 °C was 32 min, a 10-fold increase over the wild-type NpAS. However, the half-lives of DgAS and BtAS at 55 °C were 6.8 and 70 h, respectively (Choi et al., 2019; Seo et al., 2008). Although the thermostability of BtAS was higher than that of DgAS, it was thought to be affected by the amount of the enzyme; the higher the protein concentration, the greater the thermal stability of the protein (Fágáin, 1995). The specific activity of DgAS (8.6 unit/mg) was about 8-times higher than that of BtAS (1.1 unit/mg) (Choi et al., 2019; Seo et al., 2016); i.e., DgAS exhibits the same activity as BtAS, with a smaller amount of the enzyme. Therefore, the thermostability of BtAS seems to be higher as it has a higher protein concentration than DgAS. The melting temperatures of a proteins are used to compare the thermal stability per unit of protein (Pace, 1990). Although the melting temperature of BtAS was not measured, the meting temperature of DgAS was 61.4 °C higher than that of other microbial ASs (Seo et al., 2016). Although NpAS has low thermostability, it has the best ability to synthesize amylose-like polymers using sucrose as the sole substrate, compared to other microbial ASs (Seo et al., 2016). This was evidenced by the finding that DgAS synthesized 6.97 mg/mL of amylose, while NpAS synthesized 22.81 mg/mL using 100 mM sucrose. Because of these properties, NpAS has been more actively studied for starch modification capabilities, than other microbial AS. A variety of applications (starch modification, sucrose isomer production, and transglycosylation reactions for various functional compounds) using microbial AS, are described below. We first tried to understand the mechanisms of AS using the previous analyses of its amino acid sequence, its three-dimensional structure, and mutagenesis.

**Table 1:** Gene information and enzymatic properties of various microbial AS

Enzyme	Source microorganism	Genbank accession number		Optimal pH and temperature properties			Oligomeric State	References
		Gene locus tag	Protein ID	Opt. Temp. (°C)	Opt. pH	T_m_ (°C)		
AaAS	*Altermonas addita* KCTC 12195	AB469415.1	BAG82877.1	Non-AS activity			N.D	Ha et al. (2009)
AcAS	*Arthrobacter chlorophenolicus* A6	CP001341.1	ACL41561.1	45	8.0	42.6	Monomer	Seo et al. (2012a)
AmAS	*Altermonas macleodii* KCTC 2957	AB469558.1	BAG82876.1	45	8.0	48.1	N.D	Ha et al. (2009)
BtAS	*Bifidobacterium thermophilum* ATCC 25525	BTHE_RS02440	WP_044279707.1	50	6.0	N.D	N.D	Choi et al. (2019)
CcAS	*Cellulomonas carboniz* T26	AXCY01000026.1	KGM11272.1	40	7.0	47.8	Monomer	Wang et al. (2017)
DgAS	*Deinococcus geothermalis* DSM 11300	CP000359.1	ABF44874.1	50	8.0	61.4	Dimer	Seo et al. (2008)
DrAS	*Deinococcus radiodurans* ATCC 13939	NC_001263.1	NP_294657.1	50	8.0	N.D	Dimer	Pizzut-Serin et al. (2005)
DrpAS	*Deinococcus radiopugnans* ATCC 19172	MK766972	QCT05769	40	8.0	50.7	Dimer	Kim et al. (2014c; 2014d)
MaAS	*Methylomicrobium alcaliphilum* 20Z	FO082060.1	CCE22312.1	30	8.0	N.D	Monomer	But et al. (2015)
MfAS	*Methylobacillus flagellatus* KT	CP000284.1	ABE50875.1	45	8.5	50.6	Dimer	Jeong et al. (2014)
NpAS	*Neisseria polysaccharea* ATCC 43768	AJ011781.1	CAA09772.1	35	8.0	51.5	Monomer	Potocki de Montalk et al. (1999)
NsAS	*Neisseria subflava* ATCC 49275	NEISUBOT_05048	EFC51554.1	45	8.0	N.D	N.D	Park et al. (2018a)
SyAS	*Synechococcus* sp. PCC 7002	FXWN01000001.1	SMQ77851.1	30	6.5	N.D	N.D	Perez-Cenci and Salerno (2014)

*N.D* Not determined

## Amino acid sequence analysis of microbial AS based on its three-dimensional structure

**Fig. 1:**
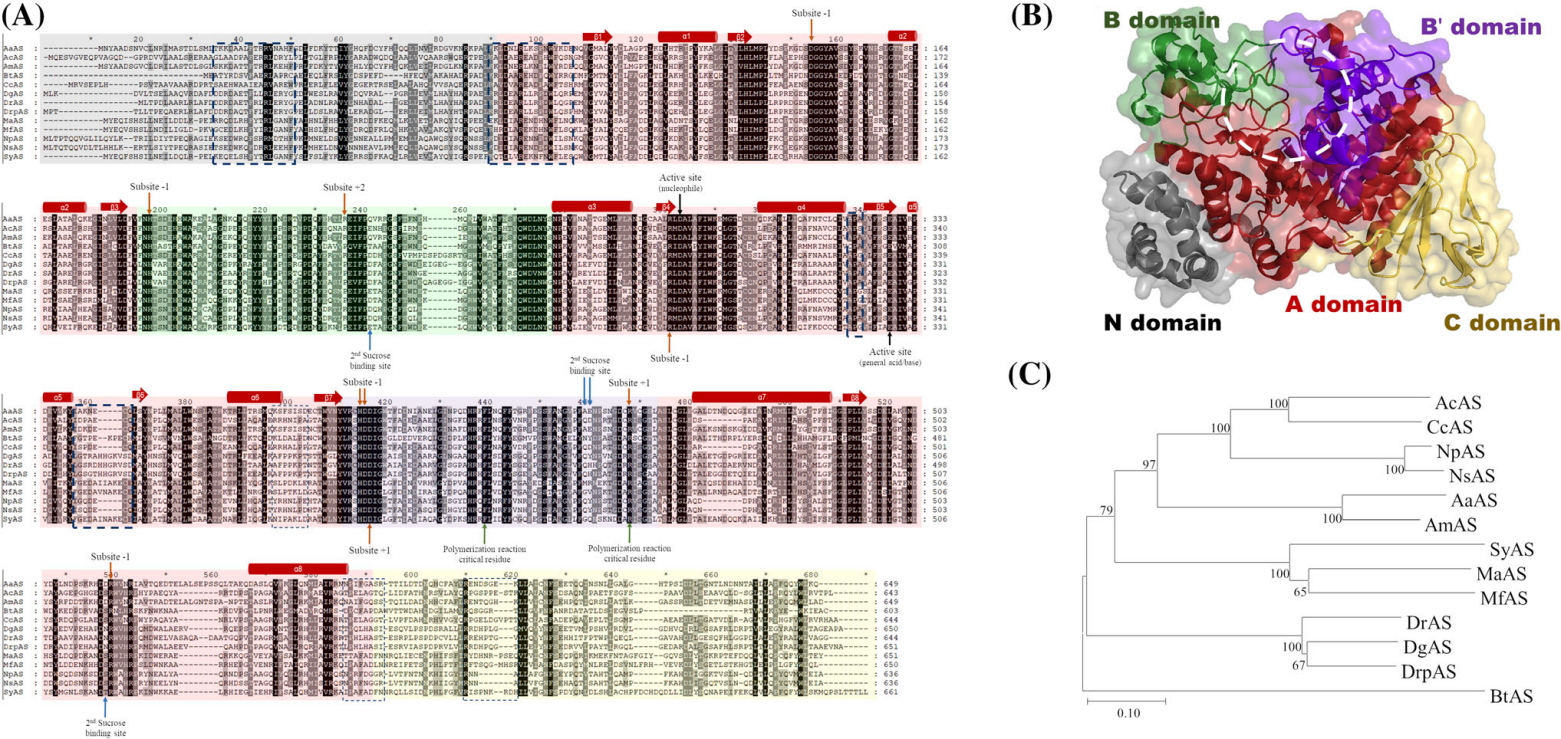
(A) The deduced amino acid sequences for various microbial AS. The 3 conserved regions related to the dimerization are surrounded by dotted boxes. (B) The three-dimensional architecture of AS. The secondary and three-dimensional structures are based on NpAS (PDB code: 1G5A), and the active pocket is indicated by a white dotted circle. (C) The phylogenetic analysis of microbial AS. The same colors are used throughout the figure to indicate the different structures: domain N (gray), domain A (red), domain B (green), domain B′ (purple), domain C (yellow)

The three-dimensional structure of NpAS was first solved among various microbial ASs (Skov et al., 2001). However, the NpAS amino acid sequence of the crystal structure (lacking the 8 amino acid sequences), differs from the first amino acid sequence determined, because the N-terminal portion of the NpAS did not dissolve. Researchers have assigned amino acid numbers to the NpAS based on the 3D-structure, using crystal structure numbering (Skov et al., 2001; Skov et al., 2002). In this review, we use the crystal structure numbering to avoid any confusion with previous studies. The 3D-structures of DrAS and DgAS have also been determined, so that there are currently three 3D AS structures (Guérin et al., 2012; Skov et al., 2013). AS has N, A, B, and C domains that are structurally identical to the domains present in the GH family 13; additionally AS has a special domain called the B′ domain (Skov et al., 2001) (Fig. 1). At this time, NpAS, which has been studied for the longest time has been subjected to the most number of protein structural analyses, was used as a reference amino acid sequence (Albenne et al., 2004; Potocki de Montalk et al., 1999). The homology of the N, A, B, B′, and C domains of the microbial ASs to NpAS are shown in Table 2. The AS showed the lowest homology between the N and C domains, and their role is still unknown (Skov et al., 2002). Some studies have shown that parts of the N and C domains affect the oligomeric state of the microbial AS (Guérin et al., 2012) (Fig. 1). Three of the ASs from the *Deinococcus* species (DgAS, DrAS, and DrpAS) and the MfAS, form dimers, whereas AcAS, CcAS, MaAC, and NpAS exist in monomer form (Table 1). The B and B′ domains are located between the A domains of the AS, and the A domain forms a (β/α)_8_-barrel fold (Fig. 1). The A, B, and B′ domains form an active-pocket that has been indicated to be the activity site of AS (Skov et al., 2002). Active-pocket architecture is also shown in other GH family 13 enzymes, such as oligo-1,6-glucosidase, β-amylase, and glucoamylase (Horvathova et al., 2001). A salt bridge formed by Arg509 and Asp144 provides topology to the active pocket in NpAS (Skov et al., 2001). The inactive NpAS variant (NpAS-E328Q) has been co-crystallized with maltoheptaose or sucrose (Jensen et al., 2004; Mirza et al., 2001). It provides valuable information about the binding interactions between the ligands and amino acid residues in AS. There are two active sites (nucleophile Asp286 and general acid/base Glu328 of NpAS) and subsites − 1 to + 6, that bind to the substrate in the active site of AS. Subsites − 1 and + 1 are catalytic sites that bind to the substrates of the AS, subsite − 1 (Asp144, His187, Arg284, His392, Asp393, and Arg509 of NPAS) is the glucosyl moiety of sucrose, and subsite + 1 (Arg394 and Asp446 of NPAS) is the binding site for the fructosyl moiety of sucrose, and the glucosyl moiety of maltooligosaccharide (Skov et al., 2002). In the crystal structure, glucose was observed to be in the β-anomer form, and it was in a ^4^C_1_ conformation at the − 1 subsite, of the active site of the NpAS (Mirza et al., 2001). AS catalyzes the hydrolysis of the glycosidic bond in sucrose (α-D-glucopyranosyl-(1 → 2)-β-d-fructofuranoside), leading to the release of fructose and formation of the glucosyl-AS intermediate (Jensen et al., 2004). Glucose is released when H_2_O reacts with glucosyl-AS and then the ‘hydrolysis reaction’ of AS occurs. When the released glucose—as the acceptor molecule maltooligosaccharide (G_n_)—binds to the + 1 subsite of the glucosyl-AS intermediate instead of water, it binds to the acceptor substance in its α-anomer form. This reaction is called the ‘transglycosylation reaction’ of AS. The maltose synthesized by AS (G_n+1_) is used again as an acceptor material to produce maltooligosaccharide (G_n+2_) with one additional glucose unit in the reaction system. Thus, the non-processive reaction of AS is to synthesize longer-lengths of maltooligosaccharides; this is called the ‘polymerization reaction’ of AS. The initial released fructose can be used as an acceptor molecule, and AS produces turanose and trehalulose. This is called the ‘isomerization reaction’ of AS, and is not observed at the initial stage of the enzyme reaction (Potocki de Montalk et al., 2000a). The DgAS produces an equivalent amount of turanose and trehalulose, different from what was found with NpAS. It was proposed that sucrose isomer production is controlled by amino acid residues from subsite + 1 (Guérin et al., 2012). The DrAS is found to have an unusual open active-pocket topology, in contrast to previous studies (Skov et al., 2013). The amino acid residues for the microbial ASs are well conserved, as not only the two essential active sites, but also the amino acid residues of the binding sites of the substrates, were consistent with those of AS (Fig. 1A). Previous studies demonstrated that residues, Arg446 and Phe417 in the B′- domain of NpAS might be present to aid the polymerization activity of AS (Pizzut-Serin et al., 2005). All the microbial ASs had two conserved residues (Phe417 and Arg446 in NpAS). Subsite + 2 in the B-domain of the microbial AS was observed to be occupied by an arginine or proline (Fig. 1A). NpAS R226A showed a 3-fold increase in insoluble amylose-like polymer formations, compared with the WT NpAS, but it was found to be less stable than the WT NpAS (Albenne et al., 2004). We constructed DgAS P219A based on this study, but the activity of the DgAS P219A was not significantly different from that of the WT DgAS (Seo et al., 2019). Liu et al, proposed that it contributes to the high stability of the DgAS, as the P219 in DgAS is smaller than the R226 in NpAS (Liu et al., 2012). Thus, they predicted that the NpAS R226P mutant, with low folding free energy, had higher thermal stability than the WT NpAS, using computer simulations. Interestingly, BtAS, DgAS, DrpAS, and MfAS containing prolines at subsite + 2 showed higher thermal stability than the NpAS. However, prolines in the DrAS and MaAS subsite + 2 displayed similar or low thermal stability, compared with the NpAS. Therefore, further research on the role of the subsite + 2 in microbial AS is needed. The NpAS exhibits atypical kinetic behavior when conformation changes occur above a critical sucrose concentration (20 mM) (Potocki de Montalk et al., 2000a). The researchers speculated that there was a 2nd sucrose binding site (Asp231, Gln437, Tyr438, and Ser508 in NpAS, SB2) on the surface of the NpAS, that resulted in the protein conformation changes at a certain sucrose concentration (Skov et al., 2002). Interestingly, DrAS showed kinetic behavior like that of NpAS (Pizzut-Serin et al., 2005), but DgAS showed typical Michaelis–Menten kinetic behavior (Seo et al., 2016). Among the residues corresponding to the SB2 of NpAS, Ser508 of NpAS was replaced by Asn519 of DgAS, and Tyr438 and Ser508 of NpAS were replaced by His433 and Asn511 of DrAS, respectively (Fig. 1A). Further, SB2 was not found in the three-dimensional structure of DrAS and DgAS (Guérin et al., 2012; Skov et al., 2013). Therefore, it is speculated that the change in kinetic behavior at critical sucrose concentrations for some microbial ASs, might not be because of SB2. In the B and B′-domains of the microbial AS, there are subsite residues to which the substrate binds and critical residues for AS activity (Fig. 1). A previous study showed that the enzyme characteristics are different according to the differences between the B and B′ domains; DgAS-B (wherein the B domain from DgAS was exchanged with the B domain of NpAS in a DgAS background) had less glucan-forming ability due to decreasing polymerization activity than DgAS, but transglycosylation activity, which could be converted to acceptor molecules, was greatly increased (Seo et al., 2016). Several laboratories are currently working to discover novel microbial AS that have improved thermostability and different acceptor specificities, by using the differences between the B and B′ domains of AS.

**Table 2:** The percentage of identity that the microbial AS domain has with NpAS

	N domain	A1 domain	B domain	A2 domain	B’ domain	A3 domain	C domain	Total	References
NpAS	100	100	100	100	100	100	100	100	Potocki de Montalk et al. (1999)
AaAS	18	51	61	61	67	32	45	47	Ha et al. (2009)
AcAS	22	63	66	77	83	57	42	58	Seo et al. (2012a)
AmAS	21	55	64	61	66	36	44	49	Ha et al. (2009)
BtAS	13	47	51	42	41	31	31	37	Choi et al. (2019)
CcAS	22	64	58	80	80	60	44	59	Wang et al. (2017)
DgAS	21	50	55	42	61	40	28	41	Seo et al. (2008)
DrAS	22	49	57	42	64	36	40	43	Pizzut-Serin et al. (2005)
DrpAS	25	50	47	42	61	42	36	42	Kim et al. (2014c; 2014d)
MaAS	16	44	55	44	45	39	35	40	But et al. (2015)
MfAS	15	40	55	39	47	42	32	38	Jeong et al. (2014)
NsAS	77	94	96	97	92	92	91	92	Park et al. (2018a)
SyAS	18	42	54	42	45	44	22	38	Perez-Cenci and Salerno (2014)

## Development of novel starches with a low digestion property: a new role for microbial AS as a starch modifier

Depending on the rate and extent of the digestion of a starch, one of the major energy sources for humans, it can be classified into one of three categories: rapidly digestible starch (RDS), slowly digestible starch (SDS), and resistant starch (RS) (Englyst et al., 1992). SDS is digested completely but slowly in the small intestine, and RS resists digestive enzymes and is fermented in the colon (Englyst et al., 2003). The physiological advantages of SDS and RS have been studied in depth (Lehmann and Robin, 2007; Sajilata et al., 2006), and they include improved management of postprandial blood glucose levels, diabetes, hypoglycemia, and hypocholesterolemia, although the exact mechanisms leading to their physiological effects differ between them (Lehmann and Robin, 2007; Sajilata et al., 2006).

According to previous studies, amylose content was significantly correlated with RS content, and slightly with SDS content (Zhang et al., 2008a), but the fine structure of amylopectin is related to the formation of SDS and RS (Ao et al., 2007; Zhang et al., 2008a; 2008b). When the ratio of short to long branched chain amylopectins is below a certain threshold, SDS formation is preferred because SDS consists mainly of imperfect crystalline regions containing small portions of double helices, as well as amorphous regions (Ao et al., 2007; Casarrubias-Castillo et al., 2012; Shin et al., 2010; 2004). However, when the ratio of short to long chains of amylopectin is above a certain threshold, the strong and perfect crystalline structures can develop through the interchain interactions of the double helices, and result in the formation of RS (Sajilata et al., 2006). The elongation of several short branch chains from external chains in amylopectin by AS, could result in the ratio of short to long chains decreasing, and that could facilitate the formation of the structures responsible for SDS and/or RS.

It was well studied that AS could extend α-1,4-linked glucan molecules, independent of the presence of α-1,6-linked glucosyl residues, by using them as acceptors. These α-1,4-linked glucan molecules could be amylose, amylopectin, and glycogen. The elongation at the non-reducing ends of the pre-existing glucan chains predominantly occurs by the consumption of glucose units released by the hydrolysis of sucrose, rather than the synthesis of new α-1,4-linked glucan polymers (Potocki de Montalk et al., 2000a; Potocki de Montalk et al., 1999; 2000a). This could provide a new perspective, and a new approach to develop starches that have low digestible properties and low glycemic indexes.

There are several studies that AS-treated starches exhibited increased RS content, compared with their respective native starches (Kim et al., 2013; 2017b; Rolland-Sabaté et al., 2004; Ryu et al., 2010). Although there are not as many studies on RS development by microbial AS, AS treatments that are able to increase the SDS content in starches have also been reported (Kim et al., 2014a; 2016a; Shin et al., 2010). The increased average length of the branched chains resulting from the decreased proportion of short chains, due to their elongation by AS, could allow for the formation of crystallites that have a slow digestible property and/or a property resistant to hydrolysis (Kim et al., 2014a; 2016a; Shin et al., 2010). Independent of the presence of amylose in starch, and amylose content if amylose is present therein, Shin et al. (2010) reported that AS increased the average branch chain length by a degree of polymerization of 13–19, but the branch chain length was increased by an approximate DP of 10, according to the findings of Ryu et al. (2010) and Kim et al. (2013). However, all of these studies listed above have found that the increases in RS content were more pronounced in the waxy starches than in the normal ones. As described above, the elongated branch chains may be the main reason for the high SDS and RS content, due to the formation of a more perfect crystalline structure via the easy association between the elongated branch chains. The fact that AS-treated starches had more perfect and ordered crystalline structures (compared with their controls) is supported by their greater thermal properties, such as melting temperature and enthalpy (Ryu et al., 2010; Shin et al., 2010; Yoo et al., 2018). It appears that AS could be a promising tool for the creation of tailored starchy foods containing a desired amount of RS.

As mentioned earlier, AS may be able to increase the ability of starches to resist digestive hydrolysis. In recent years, there have been studies to increase the slow digestible properties of the AS-treated starches, as well as their resistance to digestive hydrolysis. Via dual modifications using glycogen branching enzymes and AS, the SDS content of sweet potato starch was significantly increased. The structure of the dually treated starches may be suitable for SDS formation, by treating glycogen branching enzymes with potato starch before AS treatments (Jo et al., 2016). Another approach to increase the SDS content in AS-treated starches is to use physical treatments, such as hydrothermal treatments and temperature-cycled storage on AS-treated starches (Kim et al., 2016b; Nam et al., 2018). The high RS contents of the AS-treated starches are derived from their perfect and rigid crystalline structures, formed through the interchain interactions between the elongated branch chains of AS. The physical treatments listed above could induce the formation of SDS structures, rather than the formation of RS structures, by causing the perfect and rigid crystal structures of the AS-treated starches.

The recent utilization of AS to produce starchy food products with high SDS and RS contents, has led to the development of novel starch-based carrier materials, for functional lipid delivery systems (Kim et al., 2017a). Unmodified waxy starch was unable to form complexes with palmitic acids, whereas AS-modified starch formed complexes with palmitic acid, despite there being a small amount. Although the steric hindrance due to the highly branched structure of amylopectin may interfere the complexation, the branched chains elongated by AS were able to form complexes with fatty acids. The low digestibility of AS-treated waxy starch was maintained after complexation without any significant changes.

## Synthesis of novel functional compounds using transglycosylation activities of microbial AS

The AS transfers glucose from sucrose as a donor molecule to various hydroxylated molecules, including various glycoside compounds (arbutin, aesculin, daidzin, isoquercitin, and salicin), phenolic compounds (hydroquinone, vanillin, and zingerone), poly-phenolic compounds (aseculin, baicalein, catechin, epicatechin, luteolin, phloretin, piceid, rutin, and taxifolin), and poly-hydroxyl compounds (glycerol) (Table 3). One of the most general methods to improve the water-solubility and stability of the functional compounds is glycosylation, using various enzymes that possess transglycosylation activity (Bae et al., 2002; Li et al., 2004; Moon et al., 2006). The NpAS synthesizes two salicin glycosides, using salicin as an acceptor, and sucrose as a donor, and the transglycosylation yields achieved range from 84 to 99% (Jung et al., 2009). The synthesized salicin glycosides were confirmed as α-d-glucopyranosyl-(1 → 4)-salicin and α-d-glucopyranosyl-(1 → 4)-α-d-glucopyranosyl-(1 → 4)-salicin, by NMR analysis. In contrast, the DgAS specifically produces only one salicin glycoside, α-d-glucopyranosyl-(1 → 4)-salicin. Although the transglycosylation yield of DgAS is lower than that of NpAS, the DgAS can be employed to make a single salicin glycoside (Jung et al., 2009). Arbutin (4-hydroxyphenyl β-glucopyranoside) glycosides have been produced to improve chemical properties, such as water solubility and the whitening effect, by various enzymes (Moon et al., 2007; Park et al., 2005). The DGAS was applied to synthesize arbutin-α-glucoside in various reaction conditions (Seo et al., 2009). The maximum yield of the arbutin-α-glucoside using DgAS is determined to be over 98%, with a 1:0.5 molar ratio of donor and acceptor molecules (sucrose and arbutin, respectively), in 50 mM sodium citrate buffer pH 7 at 35 °C. The arbutin-α-glucoside is identified as 4-hydroxyphenyl β-maltoside, in which a glucose molecule is linked to arbutin via an α-(1 → 4)-glycosidic linkage (Seo et al., 2009). The whitening effect of arbutin-α-glucoside is significantly higher than that of arbutin (Jun et al., 2008). When daidzin (daidzein 7-*O*-β-d-glucoside) was reacted with DgAS and NpAS, respectively, with sucrose as a donor molecule, DgAS synthesized daidzein diglucoside and daidzein triglucoside, while NpAS synthesized only daidzein diglucoside. Furthermore, the conversion rate of DgAS is much higher than that of NpAS (Kim et al., 2019). The researchers used daidzin dissolved in DMSO, because it was insoluble in water, and 10% DMSO was present in the reaction solution. The NpAS showed very low stability in the DMSO (Emond et al., 2007), while the DgAS maintained enzyme activity in 40% DMSO (Jang et al., 2018). Therefore, the conversion of DgAS was higher than that of NpAS in the DMSO-added reaction solution, as DgAS had a higher DMSO stability than the NpAS. A whole-cell transformation method was performed to overcome the low stability of NpAS (Park et al., 2018b). *E. coli* cells that expressed NpAS were incubated with 1 mM aesculetin (6,7-dihydroxycoumarin) or aesculin (6,7-dihydroxycoumarin-6-*O*-β-glucopyranoside) as an acceptor molecule, and 10 mM sucrose as a donor molecule, with rotations of 150 rpm at 30 °C. Three transfer products (AG1, AG2, and AG3) were synthesized when using aesculetin as an acceptor, whereas only two transfer products (AGG1 and AGG2) were produced when using aesculin in the whole-cell transformation reaction. AG1 was identified as aesculetin 7-α-d-glucoside (α-cichoriin) and AGG1 was identified as 4-*O*-α-d-glycosyl aesculin, by NMR analysis. Production yields of AGG1 and AG1 with the whole-cell biotransformation method were 85 and 25%, respectively, which was significantly higher than with the free enzyme method (AGG1: 68% and AG1: 14%) (Park et al., 2018b). Cho et al, reported that the DgAS is also used to synthesize (+)-catechin-3’-*O*-α-d-glucopyranoside and (+)-catechin-3′-*O*-α-d-maltoside, using (+)-catechin and sucrose as an acceptor and donor, respectively (Cho et al., 2011). This study showed that the ratio of the acceptor to the donor is the most critical factor controlling the (+)-catechin glycosides. The transglycosylation reaction of NpAS was also carried out with catechin as well as (−)-epicatechin, as acceptor molecules (Overwin et al., 2015b). The acceptor substrate promiscuity of DgAS is explored by 21 polyphenols (Park et al., 2011). The DgAS preferentially transfers glucose to the glycoside of the polyphenol, and efficiently synthesizes glycosides for polyphenols that exist with one or more hydroxyl group, and in the z isomer form. The DgAS was employed in a bioconversion reaction to produce α-arbutin (4-hydroxyphenyl α-glucopyranoside), a powerful skin whitening agent, using sucrose as a donor and hydroquinone as an acceptor (Seo et al., 2012b). However, the bioconversion yield of α-arbutin is significantly lower (1.3%) due to the oxidation of the hydroquinone that inhibits the DgAS catalytic activity. Antioxidant agents, such as ascorbic acid, were added to the reaction mixture to prevent the oxidation of hydroquinone. Finally, a maximum bioconversion yield of α-arbutin (approximately 90%) was obtained with a 10:1 molar ratios of the donor (sucrose) and acceptor (hydroquinone) molecules in the presence of 0.2 mM ascorbic acid (Seo et al., 2012b). In contrast, The CcAS efficiently synthesized α-arbutin without using an antioxidant agent, by using 20 mM sucrose and 5 mM hydroquinone as a glucosyl donor and acceptor, respectively (Yu et al., 2018). Although the production efficiency of α-arbutin is 40–44.7%, the reaction time of CcAS is 2 h, which is a reduction in comparison with that of DgAS. The transglycosylation reaction of microbial AS has been extensively studied with a variety of flavonoids as acceptor molecules. Flavonoids have hydroxyl groups at various positions on the material and AS could react for stereospecific glucosylation at specific hydroxyl group positions. The NpAS transfected stereospecific glucosylation at the 4′-position in the B-ring of (+)-taxifolin and luteolin (these belong to flavanonol and flavone in the flavonoid, respectively) as an acceptor molecule (Malbert et al., 2014; Overwin et al., 2016). However, the bioconversion yields of (+) -taxifolin and luteolin were very low (5 and 7%, respectively). The NpAS transferred glucose from sucrose as a donor molecule to the 7 position of the A-ring in isoflavone as an acceptor molecule (Overwin et al., 2016). The conversion rate of NpAS for isoflavone was 60–70% higher than that of flavanonol and flavone. The NpAS is a catalyst for the stereospecific glucosylation of phloretin at the 4′ position (Overwin et al., 2015a). Although some NpAS variants displayed increased bioconversion efficiencies for luteolin, by 50%, DgAS showed high bioconversion yields for luetolin as an acceptor molecule (over 85%) (Jang et al., 2018; Malbert et al., 2014). The DgAS has a very efficient bioconversion efficiency for flavone compounds as an acceptor molecule. The DgAS transfers glucose to the 6-position of the A-ring of baicalein (5,6,7-trihydroxyflavone) and 6,7-dihydroxylflavone with an efficiency of 55% or more. Thus, the DgAS selectively transfers glucose to specific hydroxyl groups (the 6th position in the A-ring and 4′-position in the B-ring) of the flavone structure (Jang et al., 2018). When DgAS was used as an acceptor for various hydroxyflavones (HFVOs) and hydroxyflavanones (HFVAs), the transglycosylation reaction of the DgAS did not react with the 3-OH and 7-OH positions of HFVO and HFVA, whereas the 6-OH and 4′-OH positions exhibited strong transglycosylation reactions with DgAS (Rha et al., 2019b). Baicalein-6-α-glucoside synthesized by DgAS was compared to the baicalein, resulting in significantly increased bioavailability and stability (Kim et al., 2014b). In addition, various microbial AS were used to carry out the transglycosylation reactions for different compounds. The AmAS has glycosyltransferase activity to synthesize glucosyl piceid when piceid was used as an acceptor (Park et al., 2012). There are significant disparities between the conversion yields of enzyme reactions using purified AmAS with sucrose and piceid, and biotransformations using cultures of *E. coli* harboring the *amas* gene; the conversion yields being 35.2% and 70.8%, respectively (Park et al., 2012). Glucosyl glycerols were synthesized by the intermolecular transglycosylation activity of MfAS using glycerol as an acceptor molecule and sucrose as a donor molecule; interestingly, the production yield of 2-*O*-α-d-glucosyl-glycerol was higher than that of (2R/S)-1-*O*-α-d-glucosyl-glycerols, under all reaction conditions (Jeong et al., 2014). Chemoenzymatic synthesis that combines chemocatalysis and biocatalysis in multistep organic synthetics is a useful method for the production of novel compounds (Champion et al., 2009). Oligosaccharides mimicking the O-antigen motif of *Shigella flexneri* serotypes 1b and 3a are synthesized by chemoenzymatic synthesis using the NpAS variant that is able to glucosylate protected non-natural sugars (methyl α-L-rhamnopyranoside and allyl 2-acetamindo-2-deoxy-α-d-glucopyranoside) as acceptors.

**Table 3:** Transglycosylation reactions of various acceptor molecules using various microbial AS

Acceptor molecule	Reaction enzyme	[Donor]:[Acceptor] ratio	Reaction products	Conversion yield (%)	References
Glycoside compounds					
Arbutin	DgAS	2: 1	4-hydroxyphenyl β-maltoside	over 98.0	Seo et al. (2009)
Aseculin^a^	NpAS	10: 1	Aesculin 4-α-glucoside	85.0	Park et al. (2018b)
			Aesculin 4-α-maltoside	15.0	
Daidzin	DgAS	110: 1	Daidzein diglucoside	99.0	Kim et al. (2019)
			Daidzein triglucoside		
	NpAS	110: 1	Daidzein diglucoside	45.0	
Isoquercitrin	DgAS	14: 1	Isoquercitrin glucoside	14.6	Rha et al. (2019a)
			Isoquercitrin diglucoside	25.3	
			Isoquercitrin diglucoside isomer	11.3	
			Isoquercitrin triglucoside	46.5	
Salicin	DgAS	7: 1	α-d-glucopyranosyl-(1 → 4)-salicin	79.0	Jung et al. (2009)
			α-d-glucopyranosyl-(1 → 4)-α-d-glucopyranosyl-(1 → 4)-salicin	5.0	
	NpAS	7: 1	α-d-glucopyranosyl-(1 → 4)-salicin	15.0	
			α-d-glucopyranosyl-(1 → 4)-α-d-glucopyranosyl-(1 → 4)-salicin	84.0	
Phenolic compounds					
Hydroquinone	DgAS	10: 1: 0.2^b^	Hydroquinone α-glucopyranoside (α-arbutin)	90.0	Seo et al. (2012b)
	CcAS	4: 1	Hydroquinone α-glucopyranoside (α-arbutin)	44.7	Yu et al. (2018)
Vanillin	NpAS	1: 1	Vanillin 4-α-d-glucopyranoside	N.D	Park et al. (2011)
Zingerone	NpAS	1: 1	Zingerone 4-α-d-glucopyranoside	N.D	
Poly-phenolic compound					
Aseculetin^a^	NpAS	10: 1	Aseculetin 7-α-d- glucopyranoside (α-cichoriin)	25.0	Park et al. (2018b)
			Aseculetin 7-α-d-maltoside	2.5	
			Aseculetin 7-α-d-maltotriose	2.5	
Baicalein	DgAS	2: 1	Baicalein 6-*O*-α-d-glucopyranoside	59.1	Kim et al. (2014b)
(+)-Catechin	DgAS	1: 1	(+)-catechin-3′-*O*-α-d-glucopyranoside	97.0	Cho et al. (2011)
			(+)-catechin-3′-*O*-α-d-maltoside		
(−)-Epicatechin	NpAS	1: 1	(-)-epicatechin-3′-*O*-α-d-glucopyranoside	81.0	Overwin et al. (2015b)
			(-)-epicatechin-3′-*O*-α-d-maltoside		
			(-)-epicatechin-3′-*O*-α-d-maltotrioside		
Luteolin	DgAS	7: 1	Luteolin-4′-*O*-α-d-glucopyranoside	86.0	Jang et al. (2018)
	NpAS	29: 1	Luteolin-4′-*O*-α-d-glucopyranoside	7.0	Malbert et al. (2014)
Phloretin^a^	NpAS	15: 1	Phloretin-4′-*O*-α-d-glucopyranoside	35.0	Overwin et al. (2015a)
			Phloretin-4′-*O*-α-d-maltoside	32.0	
			Phloretin-4′-*O*-α-d-maltotrioside	28.0	
Piceid	AmAS	1: 1	Glucosyl-α-(1 → 4)-piceid	35.2 70.8^a^	Park et al. (2012)
Rutin	DrpAS	10: 1	Glucosyl-α-(1 → 4)-rutin	N.D	Kim et al. (2014c; 2014d)
(+)-Taxifolin^a^	NpAS	15: 1	(+)-taxifolin-4′-*O*-α-d-glucopyranoside	5.0	Overwin et al. (2016)
Poly-hydroxyl compounds					
Glycerol	MfAS	5: 1	2-*O*-α-d-glucosyl-glycerol	32.8	Jeong et al. (2014)
			(2R/S)-1-*O*-α-d-glucosyl-glycerol	10.2	

^a^Whole cell bioconversion reaction using recombinant *E. coli* harboring AS gene

^b^Ascorbic acid molar ratio to prevent oxidation in reaction mixture

*N.D* Not determined

## Sucrose isomer producing reactions of microbial ASs

A group of enzymes—reported from various bacterial species—such as glucansucrases, were classified into the GH13 or GH70 family, due to their capability to utilize sucrose for glucan synthesis (Lombard et al., 2013; Moulis et al., 2016). These biocatalysts often displayed two main reaction pathways, polymerization and isomerization, regardless of their bacterial sources, while the ratio of the reaction rate between α-glucan and iso-sucrose syntheses were highly dependent on the reaction conditions (Buchholz et al., 1998; Lee et al., 2017a; Park et al., 2016).

Turanose was identified fairly recently as a major reaction product from the amylosucrase of *Neisseria polysaccharea* (NpAS). It was attributed to the reaction equilibrium shift that occurred towards turanose synthesis at the expense of glucan production, by modulating the composition of reaction mixtures (Wang et al., 2012). More specifically, two different sucrose isomers were detected by the amylosucrase reaction, which were turanose and trehalulose. In the case of the NpAS reaction, turanose was exclusively generated with very tiny amounts of trehalulose. The amylosucrase from *Deinococcus geothermalis* showed equimolar production of these two sucrose isomers (Guérin et al., 2012). Most of the studies for efficient turanose production have described the biocatalytic process of applying various substrates and enzymes (Chiba and Shimomura, 1971; Shibuya et al., 2004). In addition, reagent grade turanose was produced by partial acidic hydrolysis of melezitose, isolated from an unusual and rare source of Turkestan Manna (Hehre, 1953). Other types of glucansucrases have been reported to produce sucrose isomers, like isomaltulose, trehalulose, and leucrose (Buchholz et al., 1998). Meanwhile, leucrose was identified from a dextransucrase reaction during dextran production. The biochemical properties of dextransucrase-type enzyme were well reported; this biocatalyst has synthesizing capabilities of dextran, consisting predominantly of α-(1,6)-linked α-d-glucopyranosyl units (Naessens et al., 2005) as well as sucrose isomer of leucrose (Lee et al., 2017a).

Until 2000, turanose production by the amylosucrase reaction had not been well studied and for the first time, turanose as a product was identified by Potocki de Montalk et al. (2000b). At two different concentrations of sucrose, 10 and 106 mM, the recombinant amylosucrase that originated from *N. polysaccharea* produced turanose with yields of 11 and 17%, respectively. The other sucrose isomer, trehalulose, was obtained with 0 and 2.2% yields from 10 and 106 mM of sucrose, respectively. A research group identified turanose, the major sucrose isomer, as a potential high yield bioconverted functional sweetener. There had been a clue in the previous study by Potocki de Montalk et al. (2000b), in that the increase in sucrose concentration from 10 to 106 mM resulted in significant increase in the production yields of turanose. Base on this clue, a research group identified turanose as a potential major product in the amylosucrase reaction (Wang et al., 2012). When the sucrose concentration was optimized for turanose production, the turanose yield were remarkable, resulting in more than 50% by single batch reaction. On the other hand, it was found that noticeably greater amounts of trehalulose could be synthesized by a recombinant amylosucrase that originated in *Deinococcus geothermalis* (DgAS). At 0.1 M sucrose + 0.1 M fructose, 35.2% of the glucosyl residues from sucrose were incorporated into trehalulose after the DgAS reactions, although the sucrose was not completely consumed in these reaction conditions. Thus, equivalent amounts of turanose and trehalulose were obtained by this specific amylosucrase.

Among these various reaction patterns, there is a very unique biocatalytic property expected from this enzyme that produces sucrose isomers, turanose, and trehalulose, by using fructose as a receptor molecule (Wang et al., 2012). Turanose naturally exists as a rare disaccharide in bee honey (Buchholz et al., 1998), and has approximately 50% of the sweetness of sucrose (Shibuya et al., 2004), and this sugar showed beneficial health effects of non-carcinogenicity, low-calorigenicity, and anti-obesity (Hodoniczky et al., 2012; Park et al., 2016). With these prospective uses for turanose as a novel food material, a few studies have recently investigated the bioprocesses and optimization for mass production of turanose (Park et al., 2016). As an outcome through these previous reports, the production yields of turanose became greater and maximally reach to more than 70%, by adding fructose as a reaction modulator (Wang et al., 2012).

Turanose (3-*O*-α-d-glucosyl-d-fructose) is a potential candidate to substitute for table sugar as an alternative sweetener. Toxicological studies suggested that a daily intake of 7 g/kg of turanose was not associated with any detectable acute or subchronic toxicity (Chung et al., 2017b). Turanose is considered to be a suitable sucrose replacement for patients with metabolic symptoms or disorders, because it is digested more slowly than sucrose with crude hog intestinal digesta preparation (Dahlqvist 1960) and with rat intestinal enzyme mixture (Hodoniczky et al., 2012). In addition, turanose displayed anti-obesity effects in vitro by inhibitory function on lipid accumulation, and the down-regulated lipopolysaccharide- and glucose-induced inflammation through inflammatory cytokine modulation in the Raw 264.7 macrophages (Chung et al., 2017a; Park et al., 2016).

As the sucrose substrate concentrations increased, the production titer of turanose by amylosucrase treatment also clearly increased. The maximum water solubility of sucrose has been reported to be approximately 6.13 M at 25°C (Yalkowsky and Dannenfelser, 1992). Once sucrose concentrations reached 2.5 M, the sucrose solution became very viscous and dense, and easily recrystallized and deposited on the inside walls of the glass bottle. Possible modulators of amylosucrase activity have been suggested, but fructose was the only one that can positively affect enzyme activity as an activator. It is not well known that this specific sugar directly affects the activity, as an allosteric effector, or simply works as an acceptor molecule, during transglycosylation reactions. On the contrary, it was reported that fructose could act as a competitive inhibitor against amylosucrase activity. However, this fact did not reflect any isomerization reaction that can be considered a major reaction pathway for this specific enzyme, by changing the reaction conditions (Potocki de Montalk et al., 2000a). When the interactions of fructose residues from sucrose and turanose were compared between NpAS and DgAS, it was found that the key amino acid residues located to force the fructosyl moiety to bind in an open state with the O3′, ideally positioned to explain the preference toward turanose production by NpAS. Such residues are either absent or not closely placed in DgAS. Consequentially, DgAS binds various fructo-furanosyl tautomers, and thus the interaction networks are too weak to form turanose favorably. This special geometry at catalytic subsites in DgAS is flexible, leading to other possible binding modes of fructose, and resulting in the formation of significant amounts of trehalulose (Guérin et al., 2012).

## Amylosucrase-mediated synthesis of amylose microparticles and its applications

**Fig. 2:**
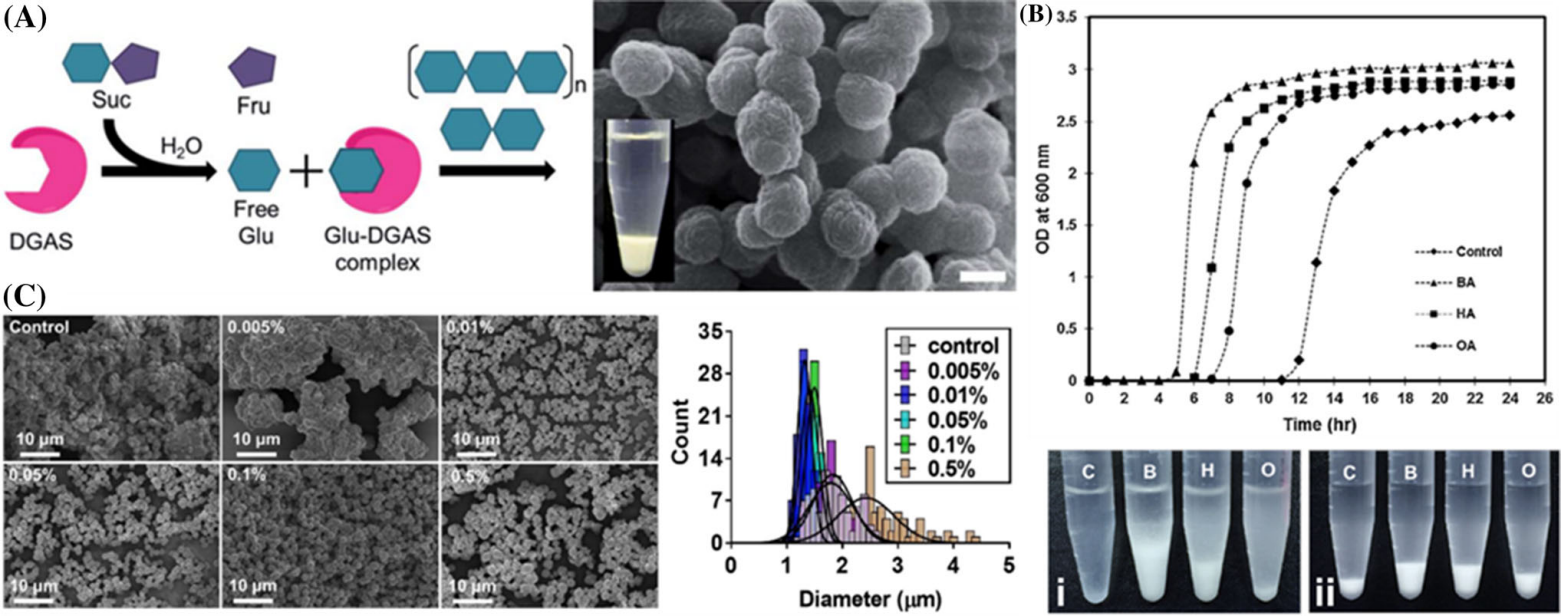
(A) Schematic illustration representing the enzymatic synthesis of AMPs and SEM image of the amyulose microparticles generated from the reaciton. The scale bar is 2 μm. (Lim et al., 2014). (B) Turbidity of the reaction solution as a function of the reaction time during the self-assembly of AMPs in the absence (control) and presence of different fatty acids (BA: butanoic acid, HA: hexanoic acid, OA: octanoic acid). Digital images of the reaction tubes corresponding to each condition after (i) 12 h and (ii) 24 h of the self-assembly reactions are shown below (Lim et al., 2016b). (c) SEM images (left) and size distribution (right) of AMPs formed with varying concentrations of lecithin from 0.005% to 0.5% (w/v) (Letona et al., 2019)

The unique catalytic activity of amylosucrase from *Deinococcus geothermalis* (DgAS) can be used to synthesize amylose microparticles. The hydrolysis of sucrose to glucose and fructose, followed by the polymerization of the glucose molecules to a linear amylose chain, with a degree of polymerization (DP) of ≈ 45, could be carried out by a single enzyme, amylosucrase (DgAS). The linear amylose chains are subsequently self-assembled into a particulate forms in an aqueous solution (Fig. 2a) (Lim et al., 2015; 2016b; 2014; Luo et al., 2018a). The self-assembly of amylose microparticles (AMPs) is a spontaneous process, since the amylose molecules in an aqueous environment are in a metastable state, due to the coexistence of hydrophobic and hydrophilic groups in the chain (Kong et al., 2014). In order to attain a lower and more stable state, the hydrophobic side of the amylose chain interacts with the adjacent chain, forming a double helix, which is then spontaneously crystallized into a lamella structure by a self-assembly process that is also called retrogradation (Buléon et al., 2007).

Understanding the self-assembly mechanism of AMPs is the key to the controlled synthesis of particles with a desired morphology, internal organization, and crystalline structure, that will determine their industrial applications. Lim et al. (2016b) demonstrated the effect of water soluble short-chain fatty acids (FAs), including butanoic, hexanoic, and octanoic acid on the amylose retrogradation, in which the rate of the self-assembly of AMPs is inversely proportional to the length of the aliphatic group of FAs (Fig. 2b). It was proposed that the carboxyl group of the FAs is responsible for the enhanced rate of the self-assembly process, via bridging double helical amylose chains to a lamella structure. The crystal structure of the resulting AMPs was not disturbed by the addition of FAs, suggesting that the FAs are eliminated from the crystal structure at the end of the self-assembly reaction. In addition, it has been reported that lecithin can act as a steric stabilizer to increase the surface charge density of amylose, and generating sufficient electrostatic repulsive forces between the adjacent solid surface of the amylose clusters, that is critical for the formation of uniform AMPs through an effective separation of the nucleation phase from the growth phase (Fig. 2c) (Letona et al., 2019).

**Fig. 3:**
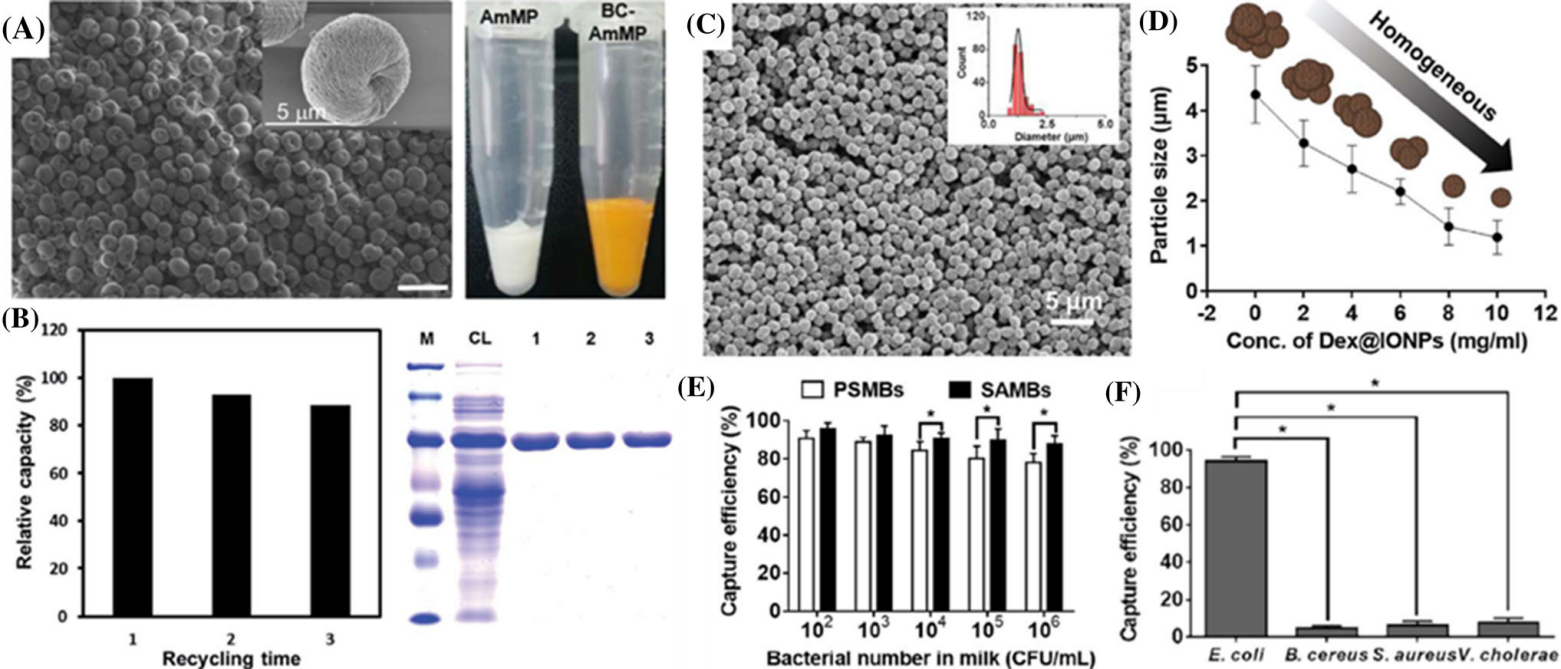
(A) SEM image of beta-carotene-encapsulated amylose microparticles. The scale bar is 10 μm (Letona et al., 2017). (B) Purification efficiency of superparamagnetic amylose microparticles (SAMPs) for target protein, MBP-GFP, from cell lysate after three rounds of recycling. The numbers (1-3) represent the number of recycling (Lim et al., 2015). (C) SEM image of SAMPs synthesized with Dex@IONPs. The inset in the upper right corner shows the size distribution of the histogram of the SAMPs. (D) The average diameter of the SAMPs formed with varying concentrations of Dex@IONPs. (E) The capture efficiency of commercial polystyrene magnetic beads (PSMBs) and superparamagnetic amylose magnetic beads (SAMBs) for target bacteria, *E. coli* O157:H7, with concentrations ranging from 10^2^ to 10^6^ CFU/mL in milk samples. (F) The capture specificity of immuno-SAMBs for target bacteria. Non-specific binding of the immuno-SAMBs with non-target bacteria was negligible (Luo et al., 2018a)

The ability of amylosucrase to produce discrete amylose microparticles has also been employed to produce biocompatible materials with various bio-applications, including drug delivery, chromatography, and bioanalytical sciences. Letona et al. (2017) reported that beta-carotene, a water-insoluble precursor of Vitamin A, could be incorporated into the AMPs by introducing the bioactive compounds during the amylosucrase-mediated self-assembly reaction (Fig. 3a). The encapsulated beta-carotenes in AMPs were found to be resistant to the environmental oxidative stresses, such as photodegradation and chemical oxidations, that are commonly taking place during processing and storage conditions. The semi-crystalline AMPs were stable enough to resist the acidic gastric environment, but slowly degraded in the intestinal environment, releasing their encapsulated bioactive compounds. These results demonstrated the potential of amylosucrase as a biocompatible carrier system for a range of bioactive compounds. Further, Lim et al. (2014) reported the synthesis of superparamagnetic amylose microparticles (SAMPs), by introducing iron oxide nanoparticles (IONPs) during the amylosucrase-mediated synthesis of amylose chains and the subsequent self-assembly reactions. The IONPs were effectively encapsulated into the growing amylose microparticles, conferring a superparamagnetic property on the synthesized amylose microparticles (Lim et al., 2016a; 2014; Luo et al., 2018a; 2018b; 2018c; 2019). The SAMPs were successfully employed to purify maltose binding protein (MBP) tagged target proteins from the bacterial cell lysate. The intrinsic affinity of the MBP tag to the surface of the SAMPs was the basis of the specific capture of the MBP-tagged target proteins. The captured target protein could easily be eluted from the SAMPs by free maltose molecules that compete for the binding site on the surface of SAMPs with the MBP-tagged target protein. The SAMPs could be recycled after stripping off the bound protein from the surface, by using free maltose (Fig. 3b). The SAMPs maintained their purification capacity of 88% after three rounds of recycling.

Luo et al. (2018a) reported that dextran-coated IONPs (Dex@IONPs) can be used as a seeding agent, rendering an epitaxial seeding effect to induce homogeneous nucleation and growth of superparamagnetic amylose microparticles (SAMPs). The resulting SAMPs were highly uniform in size and shape, and had an excellent magnetic sensitivity (Fig. 3c). It is interesting to note that the size of the SAMPs could be controlled by modulating the concentration of Dex@IONPs. The size of the SAMPs was inversely proportional to the concentration of Dex@IONPs (Fig. 3d) (Luo et al., 2018c), suggesting that the Dex@IONP acted as a seeding agent for the formation of AMMPs. Furthermore, the SAMPs were utilized to separate and concentrate target microorganisms in environmental and food samples by functionalizing the surface of SAMPs with a specific antibody. A linker protein, maltose binding protein-Streptococcal protein G (MBP-SPG), was used to conjugate an antibody to the surface of SAMP. The specific affinity of MBP and SPG towards the surface of SAMP and the Fc portion of the antibody, respectively formed the basis of the conjugation. The immuno-SAMPs were shown to have a great capture efficiency (> 90%) for the target bacteria, *E. coli* O157:H7, in food samples (Fig. 3e-f). Non-specific binding of the immuno-SAMPs with non-target bacteria was negligible due to the hydrophilic nature of the particles, demonstrating its potential as an effective material for immunomagnetic separation.

As a conclusion, AS is a versatile sucrose-hydrolyzing enzyme. There are not many enzymes that show such a diverse applications as AS. The application area is not limited to sucrose isomer production and amylose-like polymer synthesis that are direct products of AS activity from sucrose. Recently, its application is extended to the production of resistant starches, the manufacture of nanoparticles, and the synthesis of bio-functional compounds. In addition, the newly discovered AS from various microorganisms and the advanced biotechnological tools to improve the existing property of AS to enhance their thermostabilities and catalytic activities will further broaden the application range of AS.

## References

[CR1] Albenne C, Skov LK, Mirza O, Gajhede M, Feller G, D’Amico S, André G, Potocki-Véronèse G, van der Veen BA, Monsan P, Remaud-Simeon M (2004). Molecular basis of the amylose-like polymer formation catalyzed by *Neisseria polysaccharea* amylosucrase. J. Biol. Chem..

[CR2] Ao Z, Simsek S, Zhang G, Venkatachalam M, Reuhs BL, Hamaker BR (2007). Starch with a slow digestion property produced by altering its chain length, branch density, and crystalline structure. J. Agric. Food Chem..

[CR3] Bae HK, Lee SB, Park CS, Shim JH, Lee HY, Kim MJ, Baek JS, Roh HJ, Choi JH, Choe EO, Ahn DU, Park KH (2002). Modification of ascorbic acid using transglycosylation activity of *Bacillus stearothermophilus* maltogenic amylase to enhance its oxidative stability. J. Agric. Food Chem..

[CR4] Buchholz K, Noll-Borchers M, Schwengers D (1998). Production of leucrose by dextransucrase. Starch.

[CR5] Buléon A, Véronèse G, Putaux J-L (2007). Self-association and crystallization of amylose. Aust. J. Chem..

[CR6] But SY, Khmelenina VN, Reshetnikov AS, Mustakhimov II, Kalyuzhnaya MG, Trotsenko YA (2015). Sucrose metabolism in halotolerant methanotroph *Methylomicrobium alcaliphilum* 20Z. Arch. Microbiol..

[CR7] Büttcher V, Welsh T, Willmitzer L, Kossmann J (1997). Cloning and characterization of the gene for amylosucrase from Neisseria polysaccharea: production of a linear α-1,4-glucan. J. Bacteriol..

[CR8] Casarrubias-Castillo MG, Hamaker BR, Rodriguez-Ambriz SL, Bello-Pérez LA (2012). Physicochemical, structural, and digestibility properties of enzymatic modified plantain and mango starches. Starch.

[CR9] Champion E, André I, Moulis C, Boutet J, Descroix K, Morel S, Monsan P, Mulard LA, Remaud-Siméon M (2009). Design of α-transglucosidases of controlled specificity for programmed chemoenzymatic synthesis of antigenic oligosaccharides. J. Am. Chem. Soc..

[CR10] Chiba S, Shimomura T (1971). Studies on enzymatic synthesis of oligosaccharides. Agric. Biol. Chem..

[CR11] Cho HK, Kim HH, Seo DH, Jung JH, Park JH, Baek NI, Kim MJ, Yoo SH, Cha J, Kim YR, Park CS (2011). Biosynthesis of (+)-catechin glycosides using recombinant amylosucrase from *Deinococcus geothermalis* DSM 11300. Enzyme Microb. Technol..

[CR12] Choi S-W, Lee J-A, Yoo S-H (2019). Sucrose-based biosynthetic process for chain-length-defined α-glucan and functional sweetener by *Bifidobacterium* amylosucrase. Carbohydr. Polym..

[CR13] Chung J-Y, Kim Y-S, Kim Y, Yoo S-H. Regulation of inflammation by sucrose isomer, turanose, in Raw 264.7 cells. J. Cancer Prev. 22: 195-201 (2017a) 10.15430/JCP.2017.22.3.195 PMC5624461 29018785

[CR14] Chung J-Y, Lee J, Lee D, Kim E, Shin J-H, Seok PR, Yoo S-H, Kim Y (2017). Acute and 13-week subchronic toxicological evaluations of turanose in mice. Nutr. Res. Pract..

[CR15] Dahlqvist A (1960). Characterization of hog intestinal invertase as a glucosido-invertase. Acta Chem. Scand..

[CR16] Emond S, Potocki-Véronèse G, Mondon P, Bouayadi K, Kharrat H, Monsan P, Remaud-Simeon M (2007). Optimized and automated protocols for high-throughput screening of amylosucrase libraries. J. Biomol. Screen..

[CR17] Emond S, André I, Jaziri K, Potocki-Véronèse G, Mondon P, Bouayadi K, Kharrat H, Monsan P, Remaud-Simeon M (2008). Combinatorial engineering to enhance thermostability of amylosucrase. Protein Sci..

[CR18] Englyst HN, Kingman SM, Cummings JH (1992). Classification and measurement of nutritionally important starch fractions. Eur. J. Clin. Nutr..

[CR19] Englyst KN, Vinoy S, Englyst HN, Lang V (2003). Glycaemic index of cereal products explained by their content of rapidly and slowly available glucose. Br. J. Nutr..

[CR20] Fágáin CÓ (1995). Understanding and increasing protein stability. BBA-Protein Struct. Mol. Enzym..

[CR21] Guérin F, Barbe S, Pizzut-Serin S, Potocki-Véronèse G, Guieysse D, Guillet V, Monsan P, Mourey L, Remaud-Siméon M, André I, Tranier S (2012). Structural investigation of the thermostability and product specificity of amylosucrase from the bacterium *Deinococcus geothermalis*. J. Biol. Chem..

[CR22] Ha SJ, Seo DH, Jung JH, Cha J, Kim TJ, Kim YW, Park CS (2009). Molecular cloning and functional expression of a new amylosucrase from *Alteromonas macleodii*. Biosci. Biotechnol. Biochem..

[CR23] Hehre EJ, Hudson CS, Wolfrom ML (1953). The substituted-sucrose structure of melezitose. Advances in Carbohydrate Chemistry.

[CR24] Hehre EJ, Hamilton DM (1948). The conversion of sucrose to a polysaccharide of the starch-glycogen llass by *Neisseria* from the pharynx. J. Bacteriol..

[CR25] Hehre EJ, Hamilton DM, Carlson AS (1949). Synthesis of a polysaccharide of the starch-glycogen class from sucrose by a cell free, bacterial enzyme system (Amylosucrase). J. Biol. Chem..

[CR26] Hodoniczky J, Morris CA, Rae AL (2012). Oral and intestinal digestion of oligosaccharides as potential sweeteners: A systematic evaluation. Food Chem..

[CR27] Horvathova V, Janecek S, Sturdik E (2001). Amylolytic enzymes: molecular aspects of their properties. Gen. Physiol. Biophys..

[CR28] Jang S-W, Cho CH, Jung Y-S, Rha C, Nam T-G, Kim D-O, Lee Y-G, Baek N-I, Park C-S, Lee B-H, Lee S-Y, Shin HS, Seo D-H (2018). Enzymatic synthesis of α-flavone glucoside via regioselective transglucosylation by amylosucrase from *Deinococcus geothermalis*. Plos One.

[CR29] Jensen MH, Mirza O, Albenne C, Remaud-Simeon M, Monsan P, Gajhede M, Skov LK (2004). Crystal structure of the covalent Intermediate of amylosucrase from *Neisseria polysaccharea*. Biochemistry.

[CR30] Jeong J-W, Seo D-H, Jung J-H, Park J-H, Baek N-I, Kim M-J, Park C-S (2014). Biosynthesis of glucosyl glycerol, a compatible solute, using intermolecular transglycosylation activity of amylosucrase from *Methylobacillus flagellatus* KT. Appl. Biochem. Biotechnol..

[CR31] Jo AR, Kim HR, Choi SJ, Lee JS, Chung MN, Han SK, Park C-S, Moon TW (2016). Preparation of slowly digestible sweet potato *Daeyumi* starch by dual enzyme modification. Carbohydr. Polym..

[CR32] Jun SY, Park KM, Choi KW, Jang M, Kang H, Lee SH, Park KH, Cha J (2008). Inhibitory effects of arbutin-β-glycosides synthesized from enzymatic transglycosylation for melanogenesis. Biotechnol. Lett..

[CR33] Jung J-H, Seo D-H, Ha S-J, Song M-C, Cha J, Yoo S-H, Kim T-J, Baek N-I, Baik M-Y, Park C-S (2009). Enzymatic synthesis of salicin glycosides through transglycosylation catalyzed by amylosucrases from *Deinococcus geothermalis* and *Neisseria polysaccharea*. Carbohydr. Res..

[CR34] Kim B-S, Kim H-S, Hong J-S, Huber KC, Shim J-H, Yoo S-H (2013). Effects of amylosucrase treatment on molecular structure and digestion resistance of pre-gelatinised rice and barley starches. Food Chem..

[CR35] Kim BK, Kim HI, Moon TW, Choi SJ (2014). Branch chain elongation by amylosucrase: Production of waxy corn starch with a slow digestion property. Food Chem..

[CR36] Kim KH, Park Y-D, Park H, Moon K-O, Ha K-T, Baek N-I, Park C-S, Joo M, Cha J (2014). Synthesis and biological evaluation of a novel baicalein glycoside as an anti-inflammatory agent. Eur. J. Pharmacol..

[CR37] Kim M-D, Jung D-H, Seo D-H, Jung J-H, Seo E-J, Baek N-I, Yoo S-H, Park C-S (2014). Acceptor specificity of amylosucrase from *Deinococcus radiopugnans* and its application for synthesis of rutin derivatives. J. Microbiol. Biotechnol..

[CR38] Kim M-D, Seo D-H, Jung J-H, Jung D-H, Joe M-H, Lim S, Lee J-H, Park C-S (2014). Molecular cloning and expression of amylosucrase from highly radiation-resistant *Deinococcus radiopugnans*. Food Sci. Biotechnol..

[CR39] Kim EJ, Kim HR, Choi SJ, Park CS, Moon TW (2016). Low digestion property of amylosucrase-modified waxy adlay starch. Food Sci. Biotechnol..

[CR40] Kim JH, Kim HR, Choi SJ, Park C-S, Moon TW (2016). Production of an in vitro low-digestible starch via hydrothermal treatment of amylosucrase-modified normal and waxy rice starches and its structural properties. J. Agric. Food Chem..

[CR41] Kim HI, Kim HR, Choi SJ, Park C-S, Moon TW (2017). Preparation and characterization of the inclusion complexes between amylosucrase-treated waxy starch and palmitic acid. Food Sci. Biotechnol..

[CR42] Kim HR, Choi SJ, Park C-S, Moon TW (2017). Kinetic studies of in vitro digestion of amylosucrase-modified waxy corn starches based on branch chain length distributions. Food Hydrocolloid..

[CR43] Kim E-R, Rha C-S, Jung YS, Choi J-M, Kim G-T, Jung D-H, Kim T-J, Seo D-H, Kim D-O, Park C-S (2019). Enzymatic modification of daidzin using heterologously expressed amylosucrase in *Bacillus subtilis*. Food Sci. Biotechnol..

[CR44] Kong L, Lee C, Kim SH, Ziegler GR (2014). Characterization of starch polymorphic structures using vibrational sum frequency generation spectroscopy. J. Phys. Chem. B.

[CR45] Lee D, Lee J, Hong M-G, Lee B-H, Kim Y-M, Chang P-S, Kim Y, Yoo S-H (2017). Optimization of leucrose production by dextransucrase from Streptococcus mutans and its application as an adipogenesis regulator. J. Funct. Food..

[CR46] Lee Y-S, Woo J-B, Ryu S-I, Moon S-K, Han NS, Lee S-B (2017). Glucosylation of flavonol and flavanones by *Bacillus* cyclodextrin glucosyltransferase to enhance their solubility and stability. Food Chem..

[CR47] Lehmann U, Robin F (2007). Slowly digestible starch - its structure and health implications: a review. Trends Food Sci. Technol..

[CR48] Letona CAM, Park C-S, Kim Y-R (2017). Amylosucrase-mediated β-carotene encapsulation in amylose microparticles. Biotechnol. Prog..

[CR49] Letona CAM, Luo K, Jeong K-B, Adra HJ, Park C-S, Kim Y-R (2019). Effect of lecithin on the spontaneous crystallization of enzymatically synthesized short-chain amylose molecules into spherical microparticles. Polymers.

[CR50] Li D, Park JH, Park JT, Park CS, Park KH (2004). Biotechnological production of highly soluble daidzein glycosides using *Thermotoga maritima* maltosyltransferase. J. Agric. Food Chem..

[CR51] Lim M-C, Seo D-H, Jung J-H, Park C-S, Kim Y-R (2014). Enzymatic synthesis of amylose nanocomposite microbeads using amylosucrase from *Deinococcus geothermalis*. RSC Adv..

[CR52] Lim M-C, Lee G-H, Ngoc Huynh DT, Morales Letona CA, Seo D-H, Park C-S, Kim Y-R (2015). Amylosucrase-mediated synthesis and self-assembly of amylose magnetic microparticles. RSC Adv..

[CR53] Lim M-C, Lee G-H, Huynh DTN, Hong C-E, Park S-Y, Jung J-Y, Park C-S, Ko S, Kim Y-R (2016). Biological preparation of highly effective immunomagnetic beads for the separation, concentration, and detection of pathogenic bacteria in milk. Colloid Surf. B-Biointerfaces.

[CR54] Lim M-C, Park K-H, Choi J-H, Lee D-H, Letona CAM, Baik M-Y, Park C-S, Kim Y-R (2016). Effect of short-chain fatty acids on the formation of amylose microparticles by amylosucrase. Carbohydr. Polym..

[CR55] Liu M, Wang S, Sun T, Su J, Zhang Y, Yue J, Sun Z (2012). Insight into the structure, dynamics and the unfolding property of amylosucrases: Implications of rational engineering on thermostability. PLos One.

[CR56] Lombard V, Golaconda Ramulu H, Drula E, Coutinho PM, Henrissat B (2013). The carbohydrate-active enzymes database (CAZy) in 2013. Nucleic Acids Res..

[CR57] Luo K, Jeong K-B, Park C-S, Kim Y-R (2018). Biosynthesis of superparamagnetic polymer microbeads via simple precipitation of enzymatically synthesized short-chain amylose. Carbohydr. Polym..

[CR58] Luo K, Jeong K-B, You S-M, Lee D-H, Jung J-Y, Kim Y-R (2018). Surface-engineered starch magnetic microparticles for highly effective separation of a broad range of bacteria. ACS Sustain. Chem. Eng..

[CR59] Luo K, Jeong K-B, You S-M, Lee D-H, Kim Y-R (2018). Molecular rearrangement of glucans from natural starch to form size-controlled functional magnetic polymer beads. J. Agric. Food Chem..

[CR60] Luo K, Park K-H, Lee D-H, Hong C-E, Song Y-W, Yoo S-H, Kim Y-R (2019). Self-assembly kinetics of debranched short-chain glucans from waxy maize starch to form spherical microparticles and its applications. Colloid. Surf. B-Biointerfaces.

[CR61] Makarova KS, Omelchenko MV, Gaidamakova EK, Matrosova VY, Vasilenko A, Zhai M, Lapidus A, Copeland A, Kim E, Land M, Mavromatis K, Pitluck S, Richardson PM, Detter C, Brettin T, Saunders E, Lai B, Ravel B, Kemner KM, Wolf YI, Sorokin A, Gerasimova AV, Gelfand MS, Fredrickson JK, Koonin EV, Daly MJ (2007). *Deinococcus geothermalis*: the pool of extreme radiation resistance genes shrinks. PLoS ONE.

[CR62] Malbert Y, Pizzut-Serin S, Massou S, Cambon E, Laguerre S, Monsan P, Lefoulon F, Morel S, André I, Remaud-Simeon M (2014). Extending the structural diversity of α-flavonoid glycosides with engineered glucansucrases. Carbohydr. Polym..

[CR63] Mirza O, Skov LK, Remaud-Simeon M, Potocki de Montalk G, Albenne C, Monsan P, Gajhede M. Crystal structures of amylosucrase from *Neisseria polysaccharea* in complex with d-glucose and the active site mutant Glu328Gln in complex with the natural substrate sucrose. Biochemistry 40: 9032-9039 (2001) 10.1021/bi010706l 11467966

[CR64] Moon YH, Kim G, Lee JH, Jin XJ, Kim DW, Kim D (2006). Enzymatic synthesis and characterization of novel epigallocatechin gallate glucosides. J. Mol. Catal. B-Enzym..

[CR65] Moon Y, Nam S, Kang J, Kim YM, Lee JH, Kang HK, Breton V, Jun WJ, Park KD, Kimura A, Kim D (2007). Enzymatic synthesis and characterization of arbutin glucosides using glucansucrase from *Leuconostoc mesenteroides* B-1299CB. Appl. Microbiol. Biotechnol..

[CR66] Moulis C, André I, Remaud-Simeon M (2016). GH13 amylosucrases and GH70 branching sucrases, atypical enzymes in their respective families. Cell. Mol. Life Sci..

[CR67] Naessens M, Cerdobbel A, Soetaert W, Vandamme EJ (2005). Leuconostoc dextransucrase and dextran: production, properties and applications. J. Chem. Technol. Biotechnol..

[CR68] Nam SM, Kim HR, Choi SJ, Park C-S, Moon TW (2018). Effects of temperature-cycled retrogradation on properties of amylosucrase-treated waxy corn starch. Cereal Chem..

[CR69] Okada G, Hehre EJ (1974). New studies on amylosucrase, a bacterial α-D-glucosylase that directly converts sucrose to a glycogen-like α-glucan. J. Biol. Chem..

[CR70] Overwin H, Wray V, Hofer B (2015). Biotransformation of phloretin by amylosucrase yields three novel dihydrochalcone glucosides. J. Biotechnol..

[CR71] Overwin H, Wray V, Hofer B (2015). Flavonoid glucosylation by non-Leloir glycosyltransferases: formation of multiple derivatives of 3,5,7,3′,4′-pentahydroxyflavane stereoisomers. Appl. Microbiol. Biotechnol..

[CR72] Overwin H, Wray V, Seeger M, Sepúlveda-Boza S, Hofer B (2016). Flavanone and isoflavone glucosylation by non-Leloir glycosyltransferases. J. Biotechnol..

[CR73] Pace CN (1990). Measuring and increasing protein stability. Trends in Biotechnol..

[CR74] Park TH, Choi KW, Park CS, Lee SB, Kang HY, Shon KJ, Park JS, Cha J (2005). Substrate specificity and transglycosylation catalyzed by a thermostable β-glucosidase from marine hyperthermophile *Thermotoga neapolitana*. Appl. Microbiol. Biotechnol..

[CR75] Park H, Choi K, Park YD, Park CS, Cha J (2011). Enzymatic synthesis of polyphenol glycosides by amylosucrase. J. Life Sci..

[CR76] Park H, Kim J, Park JH, Baek NI, Park CS, Lee HS, Cha J (2012). Bioconversion of piceid to piceid glucoside using amylosucrase from *Alteromonas macleodii* deep ecotype. J. Microbiol. Biotechnol..

[CR77] Park M-O, Lee B-H, Lim E, Lim JY, Kim Y, Park C-S, Lee HG, Kang H-K, Yoo S-H (2016). Enzymatic process for high-yield turanose production and its potential property as an adipogenesis regulator. J. Agric. Food Chem..

[CR78] Park M-O, Chandrasekaran M, Yoo S-H (2018). Expression, purification, and characterization of a novel amylosucrase from *Neisseria subflava*. Int. J. Biol. Macromol..

[CR79] Park S, Moon K, Park CS, Jung DH, Cha J (2018). Synthesis of aesculetin and aesculin glycosides using engineered *Escherichia coli* expressing *Neisseria polysaccharea* amylosucrase. J. Microbiol. Biotechnol..

[CR80] Perez-Cenci M, Salerno GL (2014). Functional characterization of *Synechococcus* amylosucrase and fructokinase encoding genes discovers two novel actors on the stage of cyanobacterial sucrose metabolism. Plant Sci..

[CR81] Pizzut-Serin S, Potocki-Véronèse G, van der Veen BA, Albenne C, Monsan P, Remaud-Simeon M (2005). Characterisation of a novel amylosucrase from *Deinococcus radiodurans*. FEBS Lett..

[CR82] Potocki de Montalk G, Remaud-Simeon M, Willemot R-M, Sarçabal P, Planchot V, Monsan P. Amylosucrase from *Neisseria polysaccharea*: novel catalytic properties. FEBS Lett. 471: 219-223 (2000b) 10.1016/s0014-5793(00)01406-x 10767427

[CR83] Potocki de Montalk G, Remaud-Simeon M, Willemot RM, Planchot V, Monsan P. (1999) Sequence analysis of the gene encoding amylosucrase form *Neisseria polysaccharea* and characterization of the recombinant enzyme. J. Bacteriol. 181: 375-381 10.1128/jb.181.2.375-381.1999 PMC93388 9882648

[CR84] Potocki de Montalk G, Remaud-Simeon M, Willemot R-M, Monsan P. (2000a) Characterisation of the activator effect of glycogen on amylosucrase from *Neisseria polysaccharea*. FEMS Microbiol. Lett. 186: 103-108 10.1111/j.1574-6968.2000.tb09089.x 10779720

[CR85] Potocki-Veronese G, Putaux J-L, Dupeyre D, Albenne C, Remaud-Siméon M, Monsan P, Buleon A (2005). Amylose synthesized in vitro by amylosucrase: Morphology, structure, and properties. Biomacromolecules.

[CR86] Rha C-S, Choi J-M, Jung YS, Kim E-R, Ko MJ, Seo D-H, Kim D-O, Park C-S (2019). HHigh-efficiency enzymatic production of α-isoquercitrin glucosides by amylosucrase from *Deinococcus geothermalis*. Enzyme Microb. Technol..

[CR87] Rha C-S, Jung YS, Seo D-H, Kim D-O, Park C-S (2019). Site-specific α-glycosylation of hydroxyflavones and hydroxyflavanones by amylosucrase from *Deinococcus geothermalis*. Enzyme Microb. Technol..

[CR88] Rolland-Sabaté A, Colonna P, Potocki-Véronèse G, Monsan P, Planchot V (2004). Elongation and insolubilisation of α-glucans by the action of *Neisseria polysaccharea* amylosucrase. J. Cereal Sci..

[CR89] Ryu J-H, Lee B-H, Seo D, Baik M-Y, Park C-S, Wang R, Yoo S-H (2010). Production and characterization of digestion-resistantstarch by the reaction of *Neisseria polysaccharea* amylosucrase. Starch.

[CR90] Sajilata MG, Singhal RS, Kulkarni PR (2006). Resistant starch-a review. Compr. Rev. Food. Sci. Food Saf..

[CR91] Seo DH, Jung JH, Ha SJ, Yoo SH, Kim TJ, Cha J, Park CS, Park KH (2008). Molecular cloning of the amylosucrase gene from a moderate thermophilic bacterium *Deinococcus geothermalis* and analysis of its dual enzyme activity. Carbohydrate-active enzyme structure, function and application.

[CR92] Seo DH, Jung JH, Ha SJ, Song MC, Cha J, Yoo SH, Kim TJ, Baek NI, Park CS (2009). Highly selective biotransformation of arbutin to arbutin-α-glucoside using amylosucrase from *Deinococcus geothermalis* DSM 11300. J. Mol. Catal. B-Enzym..

[CR93] Seo DH, Choi HC, Kim HH, Yoo SH, Park CS (2012). Functional expression of amylosucrase, a glucan-synthesizing enzyme, from *Arthrobacter chlorophenolicus* A6. J. Microbiol. Biotechnol..

[CR94] Seo DH, Jung JH, Ha SJ, Cho HK, Jung DH, Kim TJ, Baek NI, Yoo SH, Park CS (2012). High-yield enzymatic bioconversion of hydroquinone to α-arbutin, a powerful skin lightening agent, by amylosucrase. Appl. Microbiol. Biotechnol..

[CR95] Seo D-H, Jung J-H, Jung D-H, Park S, Yoo S-H, Kim Y-R, Park C-S (2016). An unusual chimeric amylosucrase generated by domain-swapping mutagenesis. Enzyme Microb. Technol..

[CR96] Seo D-H, Jung J-H, Park C-S (2019). Improved polymerization activity of *Deinococcus geothermalis* amylosucrase by semi-rational design: Effect of loop flexibility on the polymerization reaction. Int. J. Biol. Macromol..

[CR97] Shibuya T, Mandai T, Kubota M, Fukuda S, Kurimoto M, Tsujisaka Y (2004). Production of turanose by cyclomaltodextrin glucanotransferase from *Bacillus stearothermophilus*. J. Appl. Glycosci..

[CR98] Shin SI, Choi HJ, Chung KM, Hamaker BR, Park KH, Moon TW (2004). Slowly digestible starch from debranched waxy sorghum starch: Preparation and properties. Cereal Chem..

[CR99] Shin HJ, Choi SJ, Park CS, Moon TW (2010). Preparation of starches with low glycaemic response using amylosucrase and their physicochemical properties. Carbohydr. Polym..

[CR100] Skov LK, Mirza O, Henriksen A, De Montalk GP, Remaud-Simeon M, Sarçabal P, Willemot R-M, Monsan P, Gajhede M (2001). Amylosucrase, a glucan-synthesizing enzyme from the α-amylase family. J. Biol. Chem..

[CR101] Skov LK, Mirza O, Sprogøe D, Dar I, Remaud-Simeon M, Albenne C, Monsan P, Gajhede M (2002). Oligosaccharide and sucrose complexes of amylosucrase: structural implications for the polymerase activity. J. Biol. Chem..

[CR102] Skov LK, Pizzut-Serin S, Remaud-Simeon M, Ernst HA, Gajhede M, Mirza O (2013). The structure of amylosucrase from *Deinococcus radiodurans* has an unusual open active-site topology. Acta Crystallogr. F-Struct. Biol. Commun..

[CR103] Wang R, Bae J-S, Kim J-H, Kim B-S, Yoon S-H, Park C-S, Yoo S-H (2012). Development of an efficient bioprocess for turanose production by sucrose isomerisation reaction of amylosucrase. Food Chem..

[CR104] Wang Y, Xu W, Bai Y, Zhang T, Jiang B, Mu W (2017). Identification of an α-(1,4)-glucan-synthesizing amylosucrase from *Cellulomonas carboniz* T26. J. Agric. Food Chem..

[CR105] Yalkowsky SH, Dannenfelser RM. Aquasol database of aqueous solubility. Vol. 189. College of Pharmacy, University of Arizona, Tucson (1992)

[CR106] Yamada M, Tanabe F, Arai N, Mitsuzumi H, Miwa Y, Kubota M, Chaen H, Kibata M (2006). Bioavailability of glucosyl hesperidin in rats. Biosci. Biotechnol. Biochem..

[CR107] Yoo HJ, Kim HR, Choi SJ, Park CS, Moon TW (2018). Characterisation of low-digestible starch fractions isolated from amylosucrase-modified waxy corn starch. Int. J. Food Sci. Technol..

[CR108] Yu S, Wang Y, Tian Y, Xu W, Bai Y, Zhang T, Mu W (2018). Highly efficient biosynthesis of α-arbutin from hydroquinone by an amylosucrase from *Cellulomonas carboniz*. Process Biochem..

[CR109] Zhang G, Ao Z, Hamaker BR (2008). Nutritional property of endosperm starches from maize mutants: A parabolic relationship between slowly digestible starch and amylopectin fine structure. J. Agric. Food Chem..

[CR110] Zhang G, Sofyan M, Hamaker BR (2008). Slowly digestible state of starch: Mechanism of slow digestion property of gelatinized maize starch. J. Agric. Food Chem..

