# Correction to: Versatile biotechnological applications of amylosucrase, a novel glucosyltransferase

**DOI:** 10.1007/s10068-020-00784-w

**Published:** 2020-07-08

**Authors:** Dong-Ho Seo, Sang-Ho Yoo, Seung-Jun Choi, Young-Rok Kim, Cheon-Seok Park

**Affiliations:** 1 Department of Food Science and Technology, College of Agriculture and Life Sciences, Jeonbuk National University, Jeonju, 54896 Republic of Korea; 2 Department of Food Science and Biotechnology, and Carbohydrate Bioproduct Research Center, Sejong University, Seoul, 05006 Republic of Korea; 3 Department of Food Science and Technology, Seoul National University of Science and Technology, Seoul, 01811 Republic of Korea; 4 Graduate School of Biotechnology and Institute of Life Science and Resources, Kyung Hee University, Yongin, 17104 Republic of Korea

## Correction to: Food Sci Biotechnol (2020) 29(1):1–16 10.1007/s10068-019-00686-6

The article “Versatile biotechnological applications of amylosucrase, a novel glucosyltransferase”, written by Dong-Ho Seo, Sang-Ho Yoo, Seung-Jun Choi, Young-Rok Kim and Cheon-Seok Park, was originally published Online First without Open Access. After publication in volume 29, issue 1, page 1–16 the author decided to opt for Open Choice and to make the article an Open Access publication. Therefore, the copyright of the article has been changed to © The Author(s) 2020 and the article is forthwith distributed under the terms of the Creative Commons Attribution 4.0 International License (http://creativecommons.org/licenses/by/4.0/), which permits use, duplication, adaptation, distribution and reproduction in any medium or format, as long as you give appropriate credit to the original author(s) and the source, provide a link to the Creative Commons license, and indicate if changes were made.

The original article has been corrected.

